# Association of chronic kidney disease with outcomes in acute stroke

**DOI:** 10.1007/s13760-020-01416-0

**Published:** 2020-07-13

**Authors:** Iong Man Tung, Raphae S. Barlas, Priya Vart, Joao H. Bettencourt-Silva, Allan B. Clark, Kittisak Sawanyawisuth, Kannikar Kongbunkiat, Narongrit Kasemsap, Somsak Tiamkao, Phyo K. Myint

**Affiliations:** 1 ACER, School of Medicine, Medical Sciences and Nutrition, Institute of Applied Health Sciences, University of Aberdeen Foresthill, Room 4.013 Polwarth Building, Aberdeen, AB25 2ZD Scotland, UK; 2 Stroke Research Group, Norwich Medical School, University of East Anglia, Norwich, NR4 7TJ UK; 3 Ambulatory Medicine Division, Department of Medicine, Faculty of Medicine, Khon Kaen University, Khon Kaen, 40002 Thailand; 4 Neurology Division, Department of Medicine, Faculty of Medicine, Khon Kaen University, Khon Kaen, 40002 Thailand; 5 North-Eastern Stroke Research Group, Khon Kaen University, Khon Kaen, 40002 Thailand

**Keywords:** Chronic kidney disease, Stroke, Ischaemic, Haemorrhagic, Stroke type, Complications, Outcome

## Abstract

Previous studies have found an association between chronic kidney disease and poor outcomes in stroke patients. However, there is a paucity of literature evaluating this association by stroke type. We therefore aimed to explore the association between CKD and stroke outcomes according to type of stroke. The data consisting of 594,681 stroke patients were acquired from Universal Coverage Health Security Insurance Scheme Database in Thailand. Binary logistic regression was used to assess the relationship of CKD and outcomes, which were as follows; in-hospital mortality, long length of stay (>3 days), pneumonia, sepsis, respiratory failure and myocardial infarction. Results: after fully adjusting for covariates, CKD was associated with increased odds of in-hospital mortality in patients with ischemic (OR 1.32; 95% CI = 1.27–1.38), haemorrhagic (OR 1.31; 95% CI = 1.24–1.39), and other undetermined stroke type (OR 1.44; 95% CI = 1.21–1.73). CKD was found to be associated with increased odds of pneumonia, sepsis, respiratory failure and myocardial infarction in ischaemic stroke. While CKD was found to be associated with increase odds of sepsis, respiratory failure, and myocardial infarction, decrease odds of pneumonia was observed in patients with haemorrhagic stroke. In other undetermined stroke type, CKD was found to only be associated with increase odds of sepsis and respiratory failure, while there is no significant association of CKD and increase or decrease odds with pneumonia and myocardial infarction. CKD was associated with poor outcomes in all stroke types. CKD should be considered as part of stroke prognosis as well as identifying at risk patient population for in-hospital complications.

## Introduction

Previous studies have demonstrated a high prevalence of chronic kidney disease (CKD) in patients with acute stroke, 36% when using modification of diet in renal disease formula, and 18% when using the Mayo Clinic Equation [1]. Furthermore, CKD has been associated with a twofold increased risk of in-hospital mortality [2]. However, the impact of CKD on outcomes on specific sub-types of acute stroke (ischaemic and haemorrhagic) remains uncertain. Given the pathophysiological differences between different stroke types, a detailed assessment of the association between CKD and stroke outcomes and complications is warranted. This may be helpful in evaluating the prognosis of patients admitted with stroke, thereby assisting clinicians in making informed decisions in the clinical care of stroke patients.

To date, a handful of studies assessing the association between CKD and stroke have presented findings according to stroke sub-types. However, the findings within these studies have not been consistent. For instance, Luo et al. reported a higher risk of death due to CKD in ischemic stroke patients compared to haemorrhagic stroke [3], while Snarska et al. reported higher risk in haemorrhagic stroke [4]. The aim of the current study is therefore to evaluate the association between CKD and stroke outcomes by stroke type. The outcomes under assessment include in-hospital mortality, increased length of hospital stay, pneumonia, sepsis, respiratory failure and myocardial infarction.

## Materials and methods

### Data collection

The dataset was acquired from Universal Coverage Health Security Insurance Scheme Database in Thailand. The Thai population is covered by three insurance schemes. The civil servant benefit system covers government employees and their dependents (~7% of the population) and the social security scheme that covers private sector employees (~13% of the population). The remaining 80% is covered by the universal coverage health security scheme. The current study consisted of 594,681 participants with acute stroke. Data were acquired from reimbursement forms using ICD codes. The data covers in-hospital patients from November 2005 up to January 2015.

Stroke diagnosis was made based on clinical features and investigations including brain imaging. Incident stroke was then identified with ICD coding from I61–I6400 and categorized into ischemic stroke (I63), haemorrhagic stroke (I61, I62) or other undetermined stroke type (I64). Patients with CKD were identified using ICD codes N18.1–N18.9, representing CKD stages 1–5, end-stage renal failure and unspecified CKD. All patients are either classified as having CKD or not having CKD, as due to the nature of data collection for the Universal Coverage Health Security Insurance Scheme Database, there is no distinctions made between the different stages of CKD. In addition, ICD code I12 (hypertensive kidney disease) was included. Outcomes of this study included in-hospital mortality, long length of stay and complications, including pneumonia (J14, J15, J18, J69), sepsis (A40, A41), respiratory failure (J96) and myocardial infarction (I21). Long length of stay was defined as a length of stay longer than the median (3 days). Twelve co-morbidities were selected including anaemia (D50, D53, D56, D58, D59), chronic ischemic heart disease (I25), arrhythmias (I48, I49), hypertension (I10), heart failure (I50), type 2 diabetic mellitus (E11), chronic obstructive pulmonary disease (COPD) (J44), dyslipidaemia (E78), alcohol related disease (F10), age related physical disability (R54), previous stroke (G81, I63) and other degenerative disease of nervous system (G31).

Characteristics of the study population were presented according to CKD status for overall population as well as by stroke type. The Chi-squared test and independent *T *test were used to compare the descriptive statistics of those with and without CKD. Using binary logistic regression, odds ratios were calculated for the association of CKD with various complications for the overall population and by three different stroke types. A total of four models were constructed to assess these associations. An unadjusted model; model A adjusting for age and sex; model B adjusting for model A plus the 12 co-morbidities mentioned above; model C adjusted for model B+ stroke type. The value of odds ratio is considered statically significant difference if the odds ratios’ confidence intervals cross 1.0. *p* values were also given to show statistical significance.

## Results

The current study included 594,681 stroke patients and 18,994 (3.2%) had CKD. Ischaemic stroke was the most common stroke type (51.5%), followed by haemorrhagic stroke (32.9%) with the remainder being made up of undetermined stroke types. The majority of patients in the study were elderly with a mean age ± SD of 63.75 ± 14.52. Within the entire sample population, 70,056 (11.8%) patients died within the hospital. Table 1 depicts the baseline characteristics of study participants. Patients with CKD had a significantly higher mean age compared to those without CKD. CKD patients were also more likely to experience an ischaemic stroke. Furthermore, CKD status was significantly associated with an increased likelihood of co-morbidities including; ischemic heart disease, arrhythmia, heart failure, hypertension, type 2 diabetic mellitus, anaemia, chronic obstructive pulmonary disease, dyslipidaemia and other degenerative disease of the nervous system. Conversely, there was a significantly higher prevalence of previous strokes, age related physical disability and alcohol related disease in those without CKD.

**Table 1:** Baseline characteristics of sample population

		All stroke type		
	Total (*n* = 594,681)	Without CKD (*n* = 575,687)	With CKD (*n* = 18,994)	*p* value
Mean age ± standard deviation	63.75 ± 14.52	63.62 ± 14.56	67.59 ± 12.59	< 0.001
Sex				0.007
Male	55.0 (327,167)	55.0 (316,899)	54.1 (10,268)	
Female	45.0 (267,514)	45.0 (258,788)	45.9 (8,726)	
Stroke type				< 0.001
Ischemic	51.5 (306,154)	51.2 (294,636)	60.6 (11,518)	
Hemorrhagic	32.9 (195,392)	33.1 (190,509)	25.7 (4.883)	
Other	15.7 (93,135)	15.7 (90,542)	13.7 (2,593)	
Co-morbidities				
Chronic ischemic heart disease	2.7 (16,167)	2.6 (14,867)	6.8 (1,300)	< 0.001
Arrhythmia	6.6 (39,115)	6.5 (37,569)	8.1 (1,546)	< 0.001
Heart failure	1.5 (8,855)	1.4 (8,063)	4.2 (792)	< 0.001
Hypertension	44.6 (265,189)	43.9 (252,739)	65.5 (12,450)	< 0.001
Type 2 diabetic mellitus	16.7 (99,367)	15.9 (91,428)	41.8 (7,939)	< 0.001
Anemia	5.4 (32,043)	4.8 (27,519)	23.8 (4,524)	< 0.001
Chronic obstructive pulmonary Disease	1.9 (11,497)	1.9 (10,988)	2.7 (509)	< 0.001
Dyslipidaemia	19.8 (117,498)	19.6 (112,961)	23.9 (4,537)	< 0.001
Alcohol related disease	1.5 (9.216)	1.6 (9,129)	0.5 (87)	< 0.001
Age related physical disability	0.6 (3,497)	0.6 (3,409)	0.5 (88)	0.022
Previous stroke	7.9 (46,957)	7.9 (45,642)	6.9 (1,315)	< 0.001
Other degenerative disease of nervous system	0.8 (4,941)	0.8 (4,689)	1.3 (252)	< 0.001
Outcomes				
In-hospital mortality	11.8 (70,056)	11.7 (67,095)	15.6 (2961)	< 0.001
Long length of stay	46.6 (277,258)	46.4 (267,316)	52.3 (9942)	< 0.001

Table 2 displays the baseline characteristics of participants by stroke type. Overall, the patterns observed are largely consistent with those in Table 1. However, there were a handful of exceptions to this trend. Among patients who suffered from a haemorrhagic stroke, there was no statistically significant difference in the incidence of previous strokes regardless of whether the patient had CKD or not. Likewise, when comparing patients with or without CKD among those who suffered from an undetermined stroke, there was no significant difference in the prevalence of arrhythmias and other disease of the nervous system. Regardless of stroke type, there was no significant difference in age related physical disability when comparing those with or without CKD.

**Table 2:** Baseline characteristics of sample population by stroke type

	Ischemic stroke		Haemorrhagic stroke		Other stroke types	
	Without CKD	With CKD	Without CKD	With CKD	Without CKD	With CKD
Mean age ± standard deviation	65.16 ± 13.75	69.15 ± 11.86***	60.26 ± 15.59	63.09 ± 13.71***	65.66 ± 13.64	69.13 ± 11.48***
Sex (male)	52.7 (155,385)	53.7 (6,188)*	60.1 (114,453)	56.7 (2,767)***	52.0 (47,061)	50.6 (1,313)
Co-morbidities						
Chronic ischemic Heart Disease	3.4 (10,156)	7.9 (908)***	1.2 (2,331)	5.0 (245)***	2.6 (2,380)	5.7 (147)***
Arrhythmia	9.6 (28,196)	10.3 (1,185)**	2.2 (4,116)	4.2 (207)***	5.8 (5,257)	5.9 (154)
Heart failure	1.8 (5,430)	4.5 (518)***	0.7 (1,425)	3.4 (167)***	4.1 (107)	4.1 (107)***
Hypertension	44.2 (130,355)	64.8 (7,468)***	46.4 (88,395)	70.2 (3,430)***	37.5 (33,989)	59.9 (1,552)***
Type 2 diabetic mellitus	20.3 (59,726)	44.4 (5,117)***	8.8 (16,702)	35.0 (1,707)***	16.6 (15,000)	43.0 (1,115)***
Anemia	5.4 (15,932)	24.8 (2,854)***	4.5 (8,511)	23.9 (1,167)***	3.4 (3,076)	19.4 (503)***
Chronic obstructive Pulmonary disease	2.2 (6,614)	2.9 (330)***	1.3 (2,543)	2.3 (111)***	2.0 (1,831)	2.6 (68)*
Dyslipidaemia	28.9 (85,259)	30.5 (3,509)***	7.3 (13,883)	11.7 (572)***	15.3 (13,819)	17.6 (456)**
Alcohol related disease	1.1 (3,321)	0.4 (50)***	2.6 (5,016)	0.7 (36)***	0.9 (792)	0.0 (1)***
Age related physical disability	0.5 (1,592)	0.5 (52)	0.4 (734)	0.3 (14)	1.2 (1,083)	0.8 (22)
Previous stroke	8.9 (26,260)	7.3 (844)***	6.7 (12,804)	6.3 (309)	7.3 (6,578)	6.2 (162)*
Other degenerative disease of nervous system	1.3 (3,702)	1.8 (213)***	0.3 (570)	0.5 (26)**	0.5 (417)	0.5 (13)
Outcomes						
In-hospital mortality	6.8 (20,039)	11.3 (1304)***	23.2 (44,106)	30.9 (1511)***	3.3 (2950)	5.6 (2447)***
Long length of stay	45.5 (134,160)	53.3 (6137)***	54.2 (103,351)	56.7 (2768)**	32.9 (29,805)	40.0 (1037)***

Entries marked with */**/*** shows the level of statistical significance

Entries that are not marked are not statistically significant and have a *p* value >0.05

*p  <  0.05, **p  < 0.01, ***p < 0.001

Table 3 depicts odds ratios quantifying the association between CKD and in-hospital mortality and long length of stay in different stroke types. After adjusting for confounders, CKD was found to be associated with an increased odds ratio of mortality in ischemic, haemorrhagic and other undetermined stroke types. Increased length of stay was only associated with ischemic and other undetermined stroke types, while haemorrhagic stroke is associated with a decreased length of stay. Table 4 depicts the odds ratio quantifying the association between CKD and post stroke complications, including pneumonia, sepsis, respiratory failure, myocardial infarction. CKD was found to be associated with increased odds of pneumonia, sepsis, respiratory failure and myocardial infarction in ischaemic stroke. While CKD was found to be associated with increase odds of sepsis, respiratory failure, and myocardial infarction, decrease odds of pneumonia was observed in patients with haemorrhagic stroke. In other undetermined stroke type, CKD was found to only be associated with increase odds of sepsis and respiratory failure, while there is no significant association of CKD and increase or decrease odds with pneumonia and myocardial infarction.

**Table 3:** Odds ratio with 95% confidence interval for the association of CKD with in-hospital mortality and length of stay in stroke patients

	In hospital mortality	*p* value	Length of stay	*p* value
All stroke types^a^				
Unadjusted	1.40 (1.35–1.46)	< 0.001	1.27 (1.23–1.30)	< 0.001
Model A	1.40 (1.35–1.46)	< 0.001	1.27 (1.23–1.31)	< 0.001
Model B	1.26 (1.21–1.31)	< 0.001	1.03 (1.00–1.06)	0.100
Model C	1.26 (1.21–1.32)	< 0.001	1.03 (0.99–1.05)	0.174
Ischaemic^b^				
Unadjusted	1.75 (1.65–1.86)	< 0.001	1.36 (1.31–1.42)	< 0.001
Model A	1.66 (1.56–1.76)	< 0.001	1.35 (1.30–1.40)	< 0.001
Model B	1.32 (1.24–1.41)	< 0.001	1.11 (1.07–1.15)	0.001
Haemorrhagic^b^				
Unadjusted	1.49 (1.40–1.58)	< 0.001	1.10 (1.04–1.17)	< 0.001
Model A	1.47 (1.38–1.56)	< 0.001	1.11 (1.04–1.17)	0.001
Model B	1.31 (1.23–1.39)	< 0.001	0.83 (0.79–0.88)	< 0.001
Other types^b^				
Unadjusted	1.77 (1.49–2.10)	< 0.001	1.36 (1.25–1.47)	< 0.001
Model A	1.68 (1.41–1.99)	< 0.001	1.33 (1.22–1.44)	< 0.001
Model B	1.44 (1.21–1.72)	< 0.001	1.13 (1.04–1.22)	0.005

^a^Variables adjusted: model A: age and sex, model B: model A + co-morbidities, model C: model B + stroke type

^b^Variables adjusted: model A: age and sex, model B: model A + co-morbidities

**Table 4:** Odds ratio with 95% confidence interval for the association of CKD with post-stroke complications in stroke patients

	Pneumonia	*p* value	Sepsis	*p* value	Respiratory failure	*p* value	Myocardial infarction	*p* value
All stroke types^a^								
Unadjusted	1.46 (1.40–1.52)	< 0.001	2.20 (2.07–2.33)	< 0.001	1.94 (1.85–2.02)	< 0.001	2.56 (2.26–2.91)	< 0.001
Model A	1.36 (1.30–1.42)	< 0.001	2.10 (1.98–2.23)	< 0.001	1.90 (1.82–1.99)	< 0.001	2.37 (2.09–2.69)	< 0.001
Model B	0.98 (0.93–1.02)	0.280	1.41 (1.32–1.50)	< 0.001	1.51 (1.44–1.58)	< 0.001	1.67 (1.46–1.90)	< 0.001
Model C	0.97 (0.93–1.02)	0.231	1.40 (1.32–1.49)	< 0.001	1.51 (1.44–1.58)	< 0.001	1.65 (1.45–1.89)	< 0.001
Ischaemic^b^								
Unadjusted	1.60 (1.51–1.69)	< 0.001	2.29 (2.13–2.46)	< 0.001	2.07 (1.95–2.20)	< 0.001	2.33 (2.00–2.70)	< 0.001
Model A	1.44 (1.36–1.52)	< 0.001	2.16 (2.01–2.33)	< 0.001	1.95 (1.83–2.07)	< 0.001	2.17 (1.86–2.52)	< 0.001
Model B	1.08 (1.02–1.15)	0.009	1.54 (1.42–1.66)	< 0.001	1.50 (1.41–1.60)	< 0.001	1.62 (1.38–1.90)	< 0.001
Haemorrhagic^b^								
Unadjusted	1.31 (1.21–1.42)	< 0.001	1.98 (1.77–2.22)	< 0.001	2.12 (1.98–2.27)	< 0.001	3.04 (2.32–3.99)	< 0.001
Model A	1.27 (1.17–1.37)	< 0.001	1.93 (1.72–2.16)	< 0.001	2.09 (1.95–2.24)	< 0.001	2.87 (2.19–3.76)	< 0.001
Model B	0.79 (0.72–0.86)	< 0.001	1.14 (1.01–1.28)	0.037	1.65 (1.53–1.77)	< 0.001	1.82 (1.36–2.43)	< 0.001
Other types^b^								
Unadjusted	1.47 (1.27–1.71)	< 0.001	2.20 (1.77–2.72)	< 0.001	2.27 (1.87–2.75)	< 0.001	2.07 (1.32–3.26)	0.002
Model A	1.34 (1.16–1.56)	< 0.001	2.07 (1.67–2.56)	< 0.001	2.15 (1.77–2.60)	< 0.001	1.96 (1.24–3.08)	0.004
Model B	1.13 (0.97–1.31)	0.126	1.57 (1.25–1.96)	< 0.001	1.60 (1.30–1.95)	< 0.001	1.40 (0.87–2.24)	0.167

^a^Variables adjusted: model A: age and sex, model B: model A + co-morbidity, model C: model B + stroke type

^b^Variables adjusted: model A: age and sex, model B: model A + co-morbidity

## Discussion

Our study demonstrated that CKD is associated with a significantly increased odds of in-hospital mortality, sepsis, respiratory failure and myocardial infarction even after adjusting for age, sex, stroke type and co-morbidities. When comparing ischemic stroke with haemorrhagic stroke, no significant difference in odds ratio for in-hospital mortality subsequent to adjustment for confounders. Similarly, undetermined stroke type did not demonstrate significantly increased odds of poor outcome after adjustment.

Our study also contained 15.7% of stroke that are classed as undermined type due to negative CT findings in the presence of clinically diagnosed stroke syndrome. In the absence of clear haemorrhage in the imaging, they are indeed ischaemic stroke and this is evident by the fact that the outcomes in this group is similar to CT/MRI confirmed ischemic stroke sub-type.

Our findings were comparable to those in previously published literature. For example, Ovbiagele et al. demonstrated that CKD was associated with an increased risk of in-hospital mortality [5]. However, Ovbiagele and colleagues observed a significantly increased risk is seen for ischemic stroke compared to haemorrhagic stroke after adjusting for risk factors using the Charlson Comorbidity index, whereas we did not. One possible reason for this discrepancy is that our model included anaemia, which has been shown to be an independent prognostic indicator for outcome of stroke [6, 7] but was not adjusted for in this study. Furthermore, previous literature has established anaemia as a poor prognostic indicator in patients with CKD as well [8]. The decrease in odds ratio after adjusting for anaemia may indicate that one of the major causes of poor outcome due to CKD may be partially due to CKD induced anaemia [9].

A previous study has shown that risk of infection, sepsis, and risk of poor outcomes due to infection, are increased in those with CKD [10, 11]. For example, a previous study has shown that 60% of patients who acquired sepsis after stroke died [12]. Our study shows that there is an increased risk of sepsis; however, in the case of haemorrhagic stroke, CKD patients appears to be protective against pneumonia. In patient with ischaemic stroke, it is associated with only a slight increase odds ratio for pneumonia. While CKD may be a risk factor to community acquired and hospital acquired pneumonia, it is not a risk factor to aspiration pneumonia. Community acquired and hospital acquired pneumonia, and aspiration pneumonia occurs as a complication of stroke [13–15], with up to a third of patients suffering from stroke suffers from pneumonia [10], regardless of CKD status. As seen in our study, increased risk of pneumonia is only apparent in ischaemic stroke, mostly likely due to an immune-depression response in patients caused by ischaemic stroke [11], making patients more vulnerable to infections. In the case of haemorrhagic stroke, it could be due to competing risk, where patients passed away due to other causes before the development of pneumonia was possible. Our study also shows a 51% increase in the odds of developing respiratory failure. Studies have shown an increase risk of venous thromboembolism and pulmonary embolism with patients who have CKD [16, 17], or with patients who had a stroke, which may lead to respiratory failure. Patients with pulmonary embolism can also be a cause for in-hospital mortality [18]. Finally, CKD is also known risk factor to cardiovascular complications [19, 20]. Our study found an association between CKD and development of a myocardial infarction, with a 65% increase in the odds. Cardiovascular diseases are a major contributor to mortality to patients who survives a stroke, with up to two-thirds of post stroke mortality attributed to vascular diseases [21].

The current study has several limitations. Of note, the prevalence of CKD is low in our study compared to other studies in this area [1, 5], and this is likely to be due to underreporting of CKD. Hence, the results we observed are more likely to be relevant to moderate to severe CKD as opposed to milder forms of CKD which is prevalent in the general population. The data does not stratify CKD by eGFR, hence we were unable to determine the severity of CKD in individual patients and thus the study was unable to determine whether a decreasing eGFR will result in worsening outcomes. The data does not include date of diagnosis other co-morbid conditions. As such, some cases of respiratory failure and myocardial infarction could be a pre-stroke event instead of a complication. No data was available for stroke severity (e.g., NIHSS), hence we were not able to examine role of stroke severity in observed findings.

Despite these limitations, this study has several strengths. Our large sample size allowed stratification of stroke types and provided a robust assessment of association between each stroke types. Due to the nature of the data, we are able to base this study on a national sized population thereby minimising selection bias. We are also able to assess a range of outcomes including pneumonia, sepsis, respiratory failure and myocardial infarction as well In-hospital mortality and length of stay.

To conclude, the presence of CKD can worsen the prognosis of a patient who suffered from a stroke, regardless of the type of stroke. As such, patients may benefit from taking CKD and renal dysfunction into account when prognosticating. Finally, better management of CKD may improve outcomes in patients with stroke. Future studies assessing the association between CKD and stroke outcomes may benefit from evaluating the impact interventions targeted at CKD on improving stroke outcomes.
